# Lineage-specific duplications of NBS-LRR genes occurring before the divergence of six *Fragaria* species

**DOI:** 10.1186/s12864-018-4521-4

**Published:** 2018-02-08

**Authors:** Yan Zhong, Xiaohui Zhang, Zong-Ming Cheng

**Affiliations:** 1 College of Horticulture, Nanjing Agricultural University, Nanjing, 210095 China; 2 School of Life Science, Nanjing University, Nanjing, 210023 China; 3 Department of Plant Sciences, University of Tennessee, Knoxville, TN 37996 USA

**Keywords:** NBS-LRR genes, *Fragaria* species, Disease resistance genes, Lineage-specific duplication, Duplication time

## Abstract

**Background:**

Plant disease resistance (*R*) genes are evolving rapidly and play a critical role in the innate immune system of plants. The nucleotide binding sites-leucine rich repeat (NBS-LRR) genes are one of the largest classes in plant *R* genes. Previous studies have focused on the NBS-LRR genes from one or several species of different genera, and the sequenced genomes of the genus *Fragaria* offer the opportunity to study the evolutionary processes of these *R* genes among the closely related species.

**Results:**

In this study, 325, 155, 190, 187, and 133 NBS-LRRs were discovered from *F. x ananassa*, *F. iinumae*, *F. nipponica*, *F. nubicola*, and *F. orientalis*, respectively. Together with the 144 NBS-LRR genes from *F. vesca*, a total of 1134 NBS-LRRs containing 866 multi-genes comprised 184 gene families across the six *Fragaria* genomes. Extremely short branch lengths and shallow nodes were widely present in the phylogenetic tree constructed with all of the NBS-LRR genes of the six strawberry species. The identities of the orthologous genes were highly significantly greater than those of the paralogous genes, while the *Ks* ratios of the former were very significantly lower than those of the latter in all of the NBS-LRR gene families. In addition, the *Ks* and *Ka*/*Ks* values of the TIR-NBS-LRR genes (TNLs) were significantly greater than those of the non-TIR-NBS-LRR genes (non-TNLs). Furthermore, the expression patterns of the NBS-LRR genes revealed that the same gene expressed differently under different genetic backgrounds in response to pathogens.

**Conclusions:**

These results, combined with the shared hotspot regions of the duplicated NBS-LRRs on the chromosomes, indicated that the lineage-specific duplication of the NBS-LRR genes occurred before the divergence of the six *Fragaria* species. The *Ks* and *Ka*/*Ks* ratios suggested that the TNLs are more rapidly evolving and driven by stronger diversifying selective pressures than the non-TNLs.

**Electronic supplementary material:**

The online version of this article (10.1186/s12864-018-4521-4) contains supplementary material, which is available to authorized users.

## Background

Plant disease resistance genes (*R* genes), important components of the innate immune systems of plants, specify particular recognition events with pathogen avirulence (*avr*) genes, which confer resistance to the invasion of viral, bacterial, fungal, oomycete, nematode and insect pathogens [1, 2]. Although the *R* genes have such a broad spectrum of resistance, they only encode five types of proteins, in which NBS-LRR (nucleotide binding sites- leucine-rich repeat) genes are the largest class of plant *R* genes [3]. The NBS-LRR genes contain an N-terminal domain, a conserved nucleotide-binding site (NBS) domain and a C-terminal variable leucine-rich repeat (LRR) domain. The NBS domain functions as the sites of ATP and GTP binding and hydrolyzation, and the LRR domain is critical for protein-protein interactions and peptide-ligand binding [4]. The NBS-LRR proteins are found to be nuclear or nuclear and cytoplasmic, and specifically recognize pathogenic effectors and trigger down-stream signal transduction pathways, such as hypersensitive response (HR) and programmed cell death [5–7]. The NBS-LRR genes can be further divided according to their N-terminal domain features: TIR-NBS-LRR genes have an N-terminal Toll/interleukin-1 receptor (TIR) domain and non-TIR-NBS-LRR genes contain a coiled-coil domain (CC), an RPW8/CC_R_ domain (RPW8, CC_R_ domain resembles RPW8 domain) or some other domain (X) [8–12]. A genome-wide investigation of the NBS-LRR genes has been conducted in *Arabidopsis thaliana*, *Oryza sativa*, *Vitis vivifera*, *Castanea mollissima*, *Actinidia chinensis*, *Fragaria vesca*, *Malus domestica*, *Pyrus bretschneideri*, *Prunus persica*, *Prunus mume*, and so on [13–18]. However, the NBS-LRR genes always vary in number among these plant genomes, because of the different scales of gene duplications in specific species to confront the rapidly changing pathogens in the environment [19, 20].

The genus *Fragaria* belongs to the family Rosaceae, has seven basic chromosomes (x = 7), and is considered to comprise one octoploid cultivated species (*F. x ananassa*, 2n = 8× = 56) and 24 wild species, containing 13 diploids (2n = 2× = 14), five tetraploids (2n = 4× = 28), one hexaploid (2n = 6× = 42), four octoploids (2n = 8× = 56) and one decaploid (2n = 10× = 70) [21, 22]. The origin of *F. x ananassa* is a natural hybridization in eighteenth century in Europe between two octoploids, the South American *F. chiloensis* and North American *F. virginiana* [23]. The cultivated *F. x ananassa* is an economically-important crop species around the world, and suffers from a variety of diseases causing heavy financial losses, including powdery mildew, leather rot and anthracnose, which prompted increased focus on the *R* genes and disease resistance breeding in strawberry crops. Wild species are widely known to be rich in broad disease resistance and have been successfully incorporated into cultivated crops through breeding and biotechnology [24]. Therefore, the recently released whole genome sequences of *F. x ananassa* and four wild species, *F.iinumae*, *F. nipponica*, *F. nubicola* and *F. orientalis*, provide an opportunity to conduct the genome-wide identification of NBS-LRR genes and uncover the evolutionary processes of these *R* genes among the *Fragaria* genomes [25] in relation to *F. vesca*, the reference strawberry species [14].

In this study, 1134 NBS-LRR genes and 184 gene families were identified in six *Fragaria* genomes, including 38 TNL gene families and 146 non-TNL gene families. In addition, our results suggested that the NBS-LRR genes duplicated before the divergence of the six species, according to the analysis of phylogenetic tree, synonymous substitutions and chromosome locations of the NBS-LRR genes among the six *Fragaria* genomes. Meanwhile, selective pressure and frequent sequence exchanges were also conducted, indicating the different evolutionary rates between TNL and non-TNL genes. Furthermore, the expression profiles of the NBS-LRR genes after pathogen infection showed that some *R* genes are especially expressed under various genetic backgrounds.

## Methods

### Identification of NBS-LRR genes

The whole genome sequences and annotations of *F.* x *ananassa*, *F. iinumae*, *F. nipponica*, *F. nubicola*, and *F. orientalis* were downloaded from the FTP site of Strawberry GARDEN (ftp://ftp.kazusa.or.jp/pub/strawberry/) [25]. The NBS-LRR genes in *F. vesca* were previously identified in our study [14]. Both BLAST and Hidden Markov Model (HMM) searches were employed to identify NBS-LRR genes in the five *Fragaria* species. The standard NB-ARC domain (PF00931) from the Pfam website (http://pfam.xfam.org/) was used as query sequence to TBLASTN against the whole-genome nucleotide coding sequences (CDSs) in each *Fragaria* species with an E-value ≤10^− 4^. In addition, the HMM profiles of the NB-ARC domain were also retrieved from Pfam and searched against the whole-genome protein sequences in each *Fragaria* species in hmmer 3.1 (http://hmmer.org) by using the default parameter settings. All of the hits obtained from BLAST and HMM searches were merged, and the redundancies were eliminated.

Pfam analysis was performed to verify the presence of NB-ARC domain and LRR motif in all non-redundant candidate hits, and SMART protein motif analysis (http://smart.embl-heidelberg.de/) was employed to improve the accuracy of LRR identification. Finally, the identified NBS-LRR genes were further examined whether they encoded TIR, RPW8 or CC domains by using Pfam and COILS (http://embnet.vital-it.ch/software/COILS_form.html).

### Multi-gene families of NBS-LRR genes and data analysis

An all-versus-all BLASTN search was processed in all the TNL and non-TNL CDSs among the six *Fragaria* genomes with an E-value of 1. Then, coverage of > 60% and identify between sequences of > 60% were used to divide the TNLs and the non-TNLs into multi-gene families, respectively.

The CDS alignment of each gene family was obtained based on aligning their protein sequences using Clustalw2.0 [26], which was used to calculate nonsynonymous substitutions (*Ka*), synonymous substitutions (*Ks*) and ratio of nonsynonymous to synonymous substitutions (*Ka*/*Ks*) using MEGA v6.06 [27], and investigate sequence exchange events through GENECONV 1.81 (https://www.math.wustl.edu/~sawyer/mbprogs/) with default option of 10,000 permutations (*P*-value < 0.05). For all gene families including three or more members, their CDS alignments were applied to detect positive selective pressure by using the following two models in the PAML4 package [28]: (1) the site model was set as model = 0, models M7 (beta) and M8 (beta-ω) (NS site = 7 8), and the critical values of chi-square test 5.991 (*p* < 0.05, *df* = 2) and 9.210 (*p* < 0.01, *df* = 2) were also applied in the LR test between M7 and M8; (2) the parameters of branch model were model = 0 and model 0 (NS site = 0).

### Phylogenetic tree of NBS-LRR genes

The nucleotide sequences of all NB-ARC domain regions were aligned with the MUSCLE program using the default settings through MEGA v6.06 [27]. Subsequently, a Maximum Likelihood (ML) phylogenetic tree was constructed using the Jukes-Cantor model of nucleotide evolution and 1000 replicates in FastTree v2.1.8 [29]. The same methods were also used to construct another two phylogenetic trees of the TNL and non-TNL gene families, respectively.

### Physical distributions of NBS-LRR genes on chromosomes

Among the six species, the detailed genome annotation was only available for the genome of *F. vesca*. To acquire the position information of the NBS-LRR genes from the other five species, a BLAST analysis was performed by using the CDSs of NBS-LRRs in the five genomes against the genome sequences of *F. vesca*. Then, each chromosome was divided into different regions based on 1 Mb, and the gene numbers were counted in each region of the chromosomes. The hotspot regions of the NBS-LRR genes were further examined among the six species by using Duncan tests (*P* < 0.05) across each chromosome.

### Heatmap of NBS-LRR genes after pathogen infection

The RNA-seq data of two *F. vesca* accessions, namely Hawaii 4 (HW) and Yellow Wonder 5AF7 (YW), infected by powdery mildew (*Podosphaera aphanis*) were obtained from ENA (http://www.ebi.ac.uk/ena/data/view/PRJEB4896) [30], including six samples, namely HW 0 (control), HW 1dai (day after infection), HW 8dai, YW 0 (control), YW 1dai and YW 8dai. The differentially expressed genes (DEGs) in HW (0 vs. 1dai, 0 vs. 8dai and 1dai vs.8dai) and YW (0 vs. 1dai, 0 vs. 8dai and 1dai vs.8dai) were analyzed using the edgeR package with |logFC| ≥ 2 and FDR ≤ 0.05.

The expression quantities of the *F. vesca* genes in response to *Phytophthora cactorum* were downloaded, including HW 0 and HW 2 dai [31]. The differentially expressed NBS-LRR genes were further screened on the basis of above-mentioned standards.

The heatmaps were drawn according to the expression profiles of filtered differentially expressed NBS-LRRs by the R project.

## Results

### Identification of NBS-LRR genes in six *Fragaria* species

According to searches for NBS-LRRs by using BLAST and HMM methods, a total of 1134 NBS-LRR genes were detected from the six *Fragaria* genomes, and 325, 155, 190, 187, and 133 NBS-LRRs from *F.* x *ananassa*, *F. iinumae*, *F. nipponica*, *F. nubicola*, and *F. orientalis*, respectively (Table 1). As expected, the octaploid *F.* x *ananassa* had the largest gene number compared with its five wild species, but less than 4-fold numbers of the NBS-LRRs from the diploid genomes. The tetraploid species, *F. orientalis*, possessed the least NBS-LRR genes among the six *Fragaria* species, instead of the second largest number. These might be attributed to the fact that the genome sizes of *F.* x *ananassa* and *F. orientalis* were underestimated during the whole genome sequencing [25]. In the four diploid *Fragaria* species, the NBS-LRR gene numbers normally ranged in a narrow scope, from 144 (*F. vesca*) to 190 (*F*. *nipponica*), because their similar genome sizes were close to the true genome values (~ 200 Mb) [25].

**Table 1:** NBS-LRR genes in six *Fragaria* genomes

Predicted protein domains	Letter code	*F. x ananassa*	*F. iinumae*	*F. nipponica*	*F. nubicola*	*F. orientalis*	*F. vesca* ^*a*^	Total
		(octaploid)	(diploid)	(diploid)	(diploid)	(tetraploid)	(diploid)	
NBS-LRR		325	155	190	187	133	144	1134
TIR-NBS-LRR	TNL	97	41	33	36	17	23	247
TIR-NBS-LRR	TNL’	86	36	30	34	16	21	223
TIR-TIR-NBS-LRR	TTNL	11	5	3	2	1	2	24
Non-TIR-NBS-LRR	Non-TNL	228	114	157	151	116	121	887
CC-NBS-LRR	CNL	73	40	43	55	21	60	292
CC-NBS-LRR	CNL’	68	37	42	49	21	48	265
RPW8-CC-NBS-LRR	*RPW8*-CNL	5	3	1	6	0	12	27
X-NBS-LRR	XNL	155	74	114	96	95	61	595
X-NBS-LRR	XNL’	149	69	114	95	95	50	572
RPW8-X-NBS-LRR	*RPW8*-XNL	6	5	0	1	0	12	24

^a^Data from Zhong et al., [14]

Among all the NBS-LRR genes in the six *Fragaria* genomes, there were more non-TNL genes (887) than TNLs (247), which were also detected in each *Fragaria* species, exhibiting significant difference between the 228, 114, 157, 151, 116, and 121 non-TNL genes and the 97, 41, 33, 36, 17, and 23 TNL genes in *F.* x *ananassa*, *F. iinumae*, *F. nipponica*, *F. nubicola*, *F. orientalis* and *F. vesca* (*t*-test, *P* < 0.05). The non-TNLs contained 292 CNL genes and 595 XNL genes, including 265 CNL’, 27 *RPW8*-CNLs, 572 XNL’ and 24 *RPW8*-XNLs (Table 1). Interestingly, the *RPW8*-CNL and *RPW8*-XNL, which had an N-terminal *RPW8* domain (Pfam ID: PF05659) and could be named as RPW8-NBS-LRR (RNL), were also previously found in the non-TNL genes from Rosaceae plants, legume species, Chinese chestnut and grape genomes [14–16, 32].

### Multi-gene families of NBS-LRR genes in six *Fragaria* species

All the NBS-LRR genes of the six *Fragaria* species were collected to detect the multi-gene families. In all, 184 gene families were found across the six *Fragaria* NBS-LRRs, containing 866 multi-genes, suggesting that 76.37% (866/1134) of all the NBS-LRR genes were included in the multi-gene families (Table 2). These gene families included 38 TNL gene families and 146 non-TNL gene families, with 185 TNL multi-genes and 681 non-TNL multi-genes, respectively, but showing similar proportions of multi-gene between TNLs (74.90%) and non-TNLs (76.78%).

**Table 2:** Classification of NBS-LRR genes in six *Fragaria* species

	*F. x ananassa*	*F. iinumae*	*F. nipponica*	*F. nubicola*	*F. orientalis*	*F. vesca*	Total
Number of single gene	80	38	46	30	45	29	268
Number of TNL single gene	26	14	5	6	6	5	62
Proportion of TNL single gene	26.80%	34.15%	15.15%	16.67%	35.29%	21.74%	25.10%
Number of non-TNL single gene	54	24	41	24	39	24	206
Proportion of non-TNL single gene	23.68%	21.05%	26.11%	15.89%	33.62%	19.83%	23.22%
Number of multi-gene	245	117	144	157	88	115	866
Proportion of multi-gene	75.38%	75.48%	75.79%	83.96%	66.17%	79.86%	76.37%
Number of gene family	184						
Number of TNL multi-gene	71	27	28	30	11	18	185
Proportion of TNL multi-gene	73.20%	65.85%	84.85%	83.33%	64.71%	78.26%	74.90%
Number of TNL gene family	38						
Average identity of TNL gene family	90.28%						
Number of non-TNL multi-gene	174	90	116	127	77	97	681
Proportion of non-TNL multi-gene	76.32%	78.95%	73.89%	84.11%	66.38%	80.17%	76.78%
Number of non-TNL gene family	146						
Average identity of non-TNL gene family	90.89%						

Although different numbers of multi-genes were identified in the six *Fragaria* genomes, there were four species with similar proportions of multi-genes around 75%, including 75.38% in *F. x ananassa*, 75.48% in *F. iinumae*, 75.79% in *F. nipponica*, and 79.86% in *F. vesca*, except the other two species, *F. nubicola* and *F. orientalis*, with the highest (83.96%) and lowest (66.17%) proportions, respectively. The similar ranges of large proportions were also detected in the TNL and non-TNL gene families of the six *Fragaria* species, ranging from 64.71% (*F. orientalis*) to 84.85% (*F. nipponica*) and 66.38% (*F. orientalis*) to 84.11% (*F. nubicola*). However, there were copy number variations among the six *Fragaria* species in each gene family. In the TNL gene families, the gene numbers ranged from 0 to 5 in each species of each family, except that family0 had a wide range from 1 to 25. Similarly, in non-TNL gene families, the range of 0 to 5 could be found in each family, except family7, 22 and 24 with the ranges from 0 to 13 (Additional file 1: Table S1).

The average identity in the NBS-LRR genes was 90.28% in TNL gene families and 90.89% in non-TNL gene families (Table 2). It was presented that the identity values of non-TNLs were significantly greater than those of TNLs (Additional file 2: Table S2, *t*-test, *P* < 0.05). Moreover, the identity values between orthologs were very significantly greater than those between paralogs in both the TNL and non-TNL gene families (*t*-test, *P* < 0.01), indicating that orthologs have undergone less divergence events than paralogs in the six *Fragaria* NBS-LRR genes.

### Phylogenetic tree of NBS-LRR genes among six *Fragaria* species

**Fig. 1:**
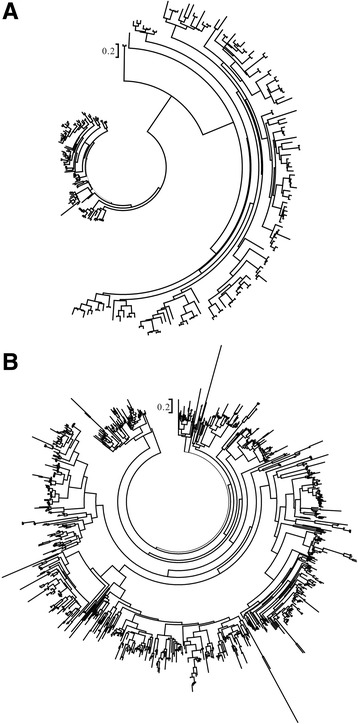
Phylogenetic tree of TNL (**a**) and non-TNL (**b**) genes among six *Fragaria* species

To further detect the evolutionary pattern of the NBS-LRR genes of the six *Fragaria* species, two unrooted phylogenetic trees of the NBS-region sequences were constructed by using FastTree software, including the TNL and non-TNL trees. For the phylogenetic tree of TNLs (Fig. 1a), it was clearly divided into two groups (group I and II) with average branch lengths 0.04 versus. 0.08 (default unit in MEGA 6), and showing highly significant difference between them (*t*-test, *P* < 0.01), which illustrated the evolutionary divergence between the two groups of *Fragaria* TNL genes. The non-TNL tree had relatively more identical branch lengths and similar topologies compared with those in the TNL tree (Fig. 1). Although the average branch length of TNL genes (0.06) was just slightly greater than that of the non-TNL genes (0.059), both the TNL-group I and II had highly significantly different branch lengths from non-TNLs, respectively (*t*-test, *P* < 0.01).

In addition, a phylogenetic tree of all the NBS-LRR genes was constructed based on the same method (Additional file 3: Figure S1), including *Fragaria* lineage-specific duplicated clades consisting of orthologs from different *Fragaria* genomes, and species-specific duplicated clades composed by clustered paralogs. A majority of the NBS-LRRs were located in *Fragaria* lineage-specific duplicated clades rather than species-specific duplicated clades, and the topological structures of genes in lineage-specific duplicated clades were in accordance with the relationship of the six *Fragaria* species (Additional file 4: Figure S2). Both the *Fragaria* lineage-specific duplicated and the species-specific duplicated clades had genes with very short branch lengths and shallow nodes, indicating that there were and few divergence events between the *Fragaria* NBS-LRR genes with high identities.

### Duplication time of NBS-LRR genes in six *Fragaria* species

To detect the duplication time of the NBS-LRR genes among the six *Fragaria* species, *Ks* values of TNLs and non-TNLs were calculated in each gene family. Considering the saturation of nucleotide substitutions, only *Ks* values lower than 1 were retained for analysis.

**Fig. 2:**
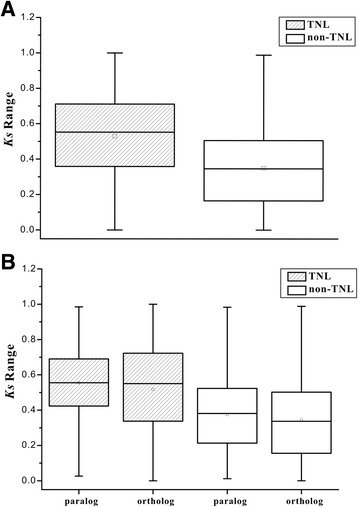
The *Ks* ranges of NBS-LRR genes in six *Fragaria* species. **a** The *Ks* ranges of TNLs and non-TNLs in the six species. **b** The *Ks* ranges between paralogs and orthologs in TNLs and non-TNLs among the six species. The bars at the top and bottom of the whiskers mean maximum and minimum values; the top and bottom of the box represent third and first quartiles; the square and bar in the box mean average and median values

On the whole, the TNL genes had greater median, mean and quartiles values than the non-TNL genes (Fig. 2a), and the *Ks* frequency of TNLs peaked at 0.3 to 0.9 greater than the peak range of 0 to 0.6 in non-TNLs (Additional file 5: Figure S3 C&F). Moreover, the *Ks* values exhibited highly significant difference between the TNLs and non-TNLs (*t*-test, *P* < 0.01), which indicated that TNL genes had very significantly higher *Ks* than non-TNL genes. In addition, the *Ks* of paralogs were highly significantly greater than those of orthologs both in TNL and non-TNL gene families (Fig. 2b, *t*-test, *P* < 0.01). The lineage-specific duplications of NBS-LRR genes occurred before the species differentiation of the six *Fragaria* plants (Fig. 2).

### Nonsynonymous and synonymous substitution of NBS-LRR genes

The ratio of nonsynonymous to synonymous nucleotide substitution (*Ka*/*Ks*) is an important parameter indicating the strength of selective constraints. Positive selection is indicated by a *Ka*/*Ks* ratio greater than 1, and neutral selection is implied by a ratio equal to 1, while purifying selection is indicated by a ratio less than 1. To detect the direction and intensity of selection, we calculated the *Ka*/*Ks* ratios in each of the TNL and non-TNL gene families among the six *Fragaria* genomes.

**Fig. 3:**
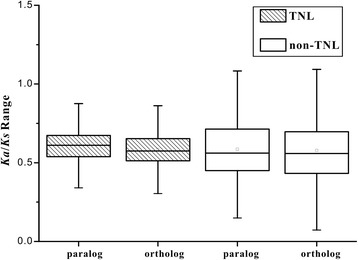
The *Ka*/*Ks* ratios of NBS-LRR genes in genomes of six *Fragaria* species

Most of the gene pairs (98%), including TNLs and non-TNLs, had *Ka*/*Ks* values less than 1 (Fig. 3), which indicated that most NBS-LRR genes were under purifying selection in the six *Fragaria* species. However, 12 and 79 gene pairs had *Ka*/*Ks* ratios greater than 1 in the TNL and non-TNL gene families, respectively, illustrating that these NBS-LRR genes were driven by positive selection. In the case of the TNL gene families, a narrower distribution of the *Ka*/*Ks* values was clearly exhibited than non-TNL gene families. Nevertheless, TNL genes had greater median and average values than those of non-TNLs, and the *Ka*/*Ks* ratios showed highly significant difference between TNLs and non-TNLs (*t*-test, *P* < 0.01). It showed that TNL genes had significantly greater *Ka*/*Ks* values than non-TNL genes, demonstrating that the TNLs are subject to stronger diversifying selection and a faster evolutionary rate than the non-TNLs.

Furthermore, the paralogs had greater median, average and quartile values than the orthologs in TNLs and non-TNLs, respectively. Especially in TNL gene families, the *Ka*/*Ks* ratios between paralogs and orthlogs displayed highly significant difference (*t*-test, *P* < 0.01), showing that paralogs had significantly greater *Ka*/*Ks* values than orthologs.

### Selective forces on NBS-LRR genes in six *Fragaria* species

To further confirm the evolutionary selective forces of NBS-LRR genes in the six *Fragaria* species, we also calculated the ω (*dN*/*dS*) and 2Δln values by branch and site models of PAML4 in TNL and non-TNL gene families. In the two model tests, 105 gene families were estimated, containing 23 TNL and 82 non-TNL gene families with three or more gene members (Tables 3 and 4). For the ω ratios, 99 gene families had average ω values less than 1, indicating purifying selection was the main force acting on the 22 TNL and 77 non-TNL gene families. One TNL and two non-TNL gene families had average ω ratios approximately equal to 1, suggesting that these gene families underwent neutral nonfunctionalization between duplicates. Moreover, three non-TNL gene families with ω ratios greater than 1 demonstrated that higher substitution rates were found in them caused by neofunctionalization [33]. In addition, LR tests were performed to detect the positive selection on amino acid sites represented by 2Δln values. Among the 23 TNL gene families, there were 15 families (65.22%) had amino acid sites under significant (2Δln > 5.991, *P* < 0.05) or highly significant (2Δln > 9.210, *P* < 0.01) positive selection. However, 81.71% of the 82 non-TNL gene families had positively selected sites examined by significance (2Δln > 5.991, *P* < 0.05) or highly significance (2Δln > 9.210, *P* < 0.01) tests. It is worth mentioning that there were 24, 45 and 92 positively selected sites distributed in TIR domains, NB-ARC domains and LRR motifs in TNLs and 26, 285 and 465 positively selected sites in CC regions, NB-ARC domains and LRR motifs in non-TNLs. It was showed that in LRR motifs had more positively selected sites than NB-ARC and other domains in these NBS-LRR genes.

**Table 3:** Positive selection and sequence exchange events of TNLs in six *Fragaria* species

Family	*Ka*/*Ks*^a^	ω (*dN*/*dS*) ^b^	2Δln^c^	LR test^c^	Sequence exchange events^d^
family0	0.60	0.23	0.00		0
family1	0.62	0.67	45.74	**	18
family2	0.77	–			0
family3	0.65	0.73	40.23	**	15
family4	0.37	0.38	0.54		1
family5	0.73	1.01	110.93	**	14
family6	0.41	0.35	1.38		0
family7	0.54	0.54	44.13	**	9
family8	0.96	–			0
family9	0.62	0.49	47.84	**	29
family10	0.75	–			0
family11	0.57	0.58	14.81	**	5
family12	0.89	–			0
family13	0.77	0.77	299.48	**	2
family14	0.51	0.49	101.49	**	5
family15	0.59	0.61	25.08	**	2
family16	0.22	–			0
family17	0.85	0.85	9.70	**	4
family18	0.79	–			0
family19	0.35	0.33	3.78		8
family20	0.47	0.42	8.82	*	7
family21	0.78	–			0
family22	0.28	0.30	0.00		1
family23	0.56	0.54	27.06	**	6
family24	0.51	0.45	4.64		3
family25	0.59	0.63	81.98	**	4
family26	0.55	–			0
family27	0.67	0.61	28.33	**	4
family28	0.58	–			0
family29	0.63	–			0
family30	0.71	–			0
family31	0.54	–			0
family32	0.71	0.62	80.84	**	3
family33	0.41	–			0
family34	0.41	0.43	2.73		0
family35	0.88	–			0
family36	0.26	0.15	0.00		0
family37	0.60	–			0

^a^Average *Ka*/*Ks* ratio of each gene family by using MEGA6; ^b^ω (*dN*/*dS*) value of each gene family was calculated by using branch model in PAML software; ^c^ 2Δln means the LR-test result using site model in PAML software; * and ** represent significant (2Δln > 5.991, *P* < 0.05) and highly significant (2Δln > 9.210, *P* < 0.01) tests for positive selection between model M7 and M8; ^d^ Sequence exchange event shows the statistically significant sequence exchange events (*P* < 0.05)

**Table 4:** Positive selection and sequence exchange events of non-TNLs in six *Fragaria* species

Family	*Ka*/*Ks* ^a^	ω (*dN*/*dS*) ^b^	2Δln ^c^	LR test ^c^	Sequence exchange events ^d^
family0	0.45	0.42	35.48	**	11
family1	0.78	0.83	59.37	**	28
family2	0.59	0.59	75.22	**	39
family3	1.46	0.99	43.01	**	0
family4	0.64	0.72	4.67		10
family5	0.93	–			0
family6	0.83	0.93	72.76	**	11
family7	0.58	0.68	73.55	**	10
family8	0.69	0.49	27.03	**	63
family9	0.63	0.54	15.19	**	5
family10	0.60	0.36	10.98	**	12
family11	0.91	0.97	62.72	**	5
family12	0.67	–			0
family13	0.82	0.87	146.39	**	77
family14	1.14	–			0
family15	0.42	0.40	0.19		4
family16	0.41	–			0
family17	–	–			0
family18	0.51	0.43	0.00		0
family19	0.65	0.65	37.48	**	3
family20	0.79	0.93	129.67	**	5
family21	0.74	0.92	10.52	**	4
family22	0.68	0.58	25.75	**	119
family23	0.64	0.58	47.88	**	2
family24	0.45	0.50	23.82	**	4
family25	0.58	0.54	111.42	**	8
family26	0.57	0.59	74.25	**	18
family27	1.00	1.09	116.83	**	26
family28	0.72	0.62	18.65	**	0
family29	0.92	–			0
family30	0.94	–			0
family31	0.73	–			0
family32	0.96	0.87	47.42	**	16
family33	1.06	0.95	98.69	**	8
family34	0.71	0.44	1.14		1
family35	0.62	–			0
family36	0.93	–			0
family37	0.65	–			0
family38	0.54	0.50	0.00		1
family39	0.50	0.55	26.57	**	4
family40	0.78	0.77	6.29	*	0
family41	0.60	–			0
family42	0.66	0.65	179.47	**	36
family43	0.42	0.52	13.77	**	0
family44	0.60	–			0
family45	0.95	–			0
family46	0.67	0.68	58.45	**	21
family47	0.31	0.34	160.78	**	5
family48	0.51	–			0
family49	0.27	–			0
family50	0.71	0.88	188.51	**	6
family51	0.47	0.44	69.96	**	15
family52	0.81	–			0
family53	0.54	–			0
family54	0.65	0.58	34.41	**	23
family55	1.70	2.23	113.38	**	1
family56	0.66	0.49	10.66	**	10
family57	0.51	0.32	0.00		2
family58	0.55	0.42	15.31	**	1
family59	0.25	0.34	18.49	**	1
family60	–	–			0
family61	0.84	–			0
family62	0.67	–			0
family63	0.17	–			0
family64	0.71	0.73	159.69	**	16
family65	0.45	0.50	8.95	*	25
family66	0.92	0.88	36.06	**	0
family67	0.44	0.15	0.00		0
family68	0.82	–			0
family69	0.55	0.32	8.34	*	1
family70	1.16	0.47	57.04	**	2
family71	0.70	–			0
family72	0.93	1.16	7.52	*	0
family73	0.99	–			0
family74	0.57	0.57	62.86	**	4
family75	0.63	0.80	4.68		0
family76	0.58	0.57	9.64	**	6
family77	0.59	0.66	21.23	**	0
family78	0.62	–			0
family79	0.57	0.55	80.40	**	11
family80	0.67	0.67	297.98	**	7
family81	0.55	0.63	15.57	**	0
family82	2.48	–			0
family83	0.87	–			0
family84	1.25	–			0
family85	0.70	0.78	48.80	**	23
family86	1.21	1.29	91.70	**	0
family87	0.99	0.91	397.48	**	6
family88	0.89	1.09	27.11	**	3
family89	0.36	0.43	0.69		0
family90	0.33	–			0
family91	0.36	0.36	7.96	*	0
family92	0.31	0.32	0.75		1
family93	0.42	–			0
family94	0.71	–			0
family95	0.72	–			0
family96	0.60	–			0
family97	0.87	0.89	54.59	**	3
family98	0.41	0.36	56.24	**	0
family99	0.58	0.54	3.18		0
family100	0.25	0.22	1.64		21
family101	0.56	0.61	9.90	**	15
family102	0.90	–			0
family103	0.77	–			0
family104	0.61	0.68	61.66	**	6
family105	0.60	0.53	20.30	**	0
family106	0.26	–			0
family107	0.70	0.68	22.56	**	18
family108	0.49	–			0
family109	0.29	0.32	0.00		0
family110	0.45	–			0
family111	0.60	0.45	14.82	**	1
family112	0.40	0.44	0.00		0
family113	0.55	0.76	12.69	**	0
family114	0.76	–			0
family115	0.37	–			0
family116	0.76	0.80	102.63	**	7
family117	0.69	0.47	9.51	**	2
family118	0.68	0.60	46.89	**	3
family119	–	–			0
family120	0.42	–			0
family121	0.24	–			0
family122	0.20	0.26	0.41		0
family123	0.50	–			0
family124	1.62	0.77	72.18	**	1
family125	0.73	0.84	9.74	**	1
family126	0.62	–			0
family127	0.34	–			0
family128	0.53	0.51	22.29	**	6
family129	0.63	–			0
family130	0.71	–			0
family131	0.70	–			0
family132	–	–			0
family133	1.06	–			0
family134	0.90	–			0
family135	0.62	–			0
family136	0.75	–			0
family137	0.75	–			0
family138	0.84	–			0
family139	0.52	–			0
family140	0.68	–			0
family141	0.69	–			0
family142	0.49	–			0
family143	0.80	–			0
family144	1.22	–			0
family145	0.53	–			0

^a^Average *Ka*/*Ks* ratio of each gene family by using MEGA6; ^b^ ω (*dN*/*dS*) value of each gene family was calculated by using branch model in PAML software; ^c^ 2Δln means the LR-test result using site model in PAML software; * and ** represent significant (2Δln > 5.991, *P* < 0.05) and highly significant (2Δln > 9.210, *P* < 0.01) tests for positive selection between model M7 and M8; ^d^ Sequence exchange event shows the statistically significant sequence exchange events (*P* < 0.05)

The sequence exchange events include gene conversion, recombination, and unequal crossing-over. In totally, 944 sequence exchange events occurred in the NBS-LRR gene families, including 140 in TNLs and 804 in non-TNLs, and 20.00% and 17.04% of sequence exchange events occurred between paralogs in TNL and non-TNL gene families, respectively. The sequence exchange events among paralogs (28 in TNLs and 137 in non-TNLs) could raise the sequence homogeneity within a species and the sequence divergence between species [34].

### Chromosomal distribution of NBS-LRR genes among the six *Fragaria* species

**Fig. 4:**
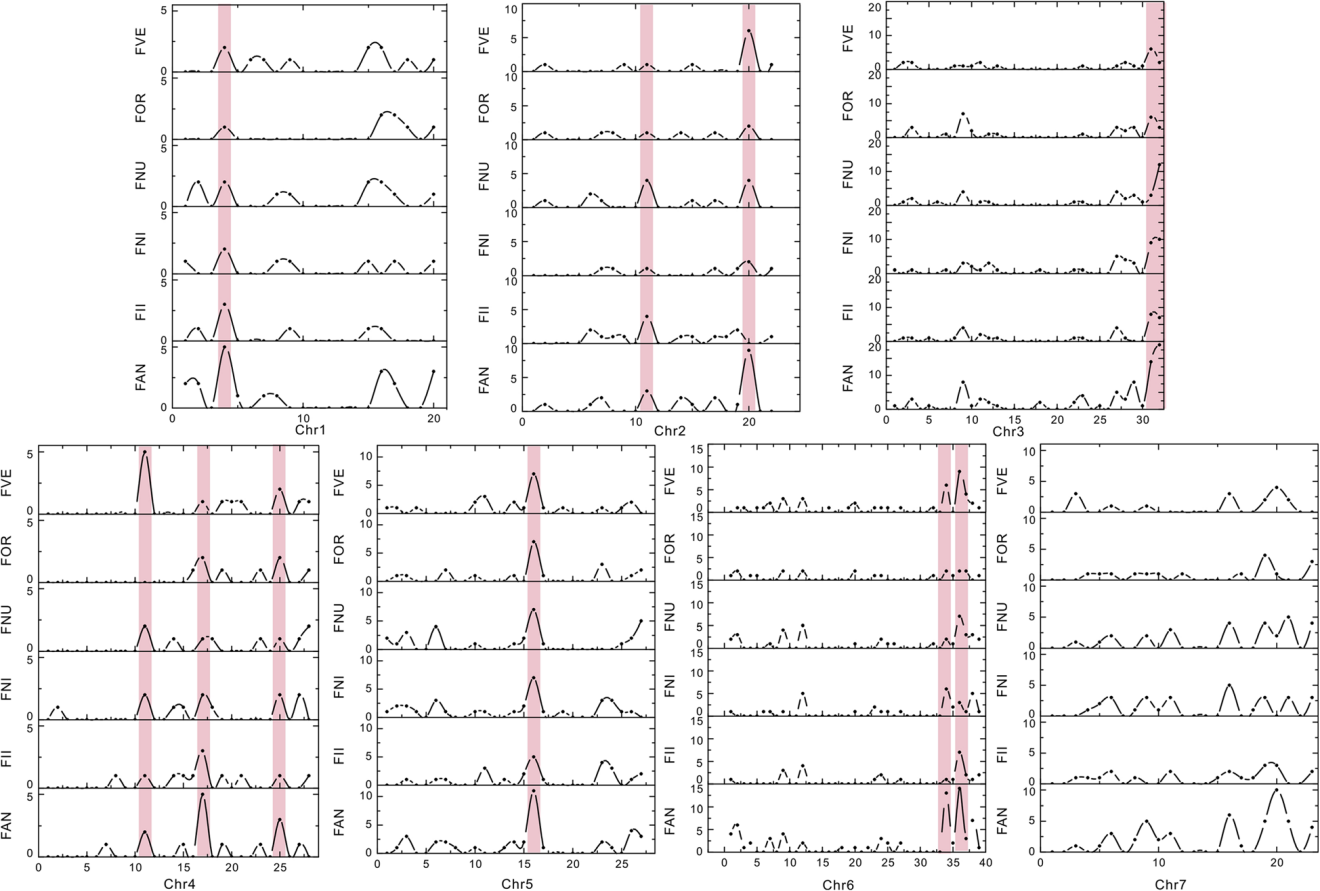
Chromosome distribution of NBS-LRR genes in six *Frageria* species. Black dots and lines represent the gene numbers of NBS-LRRs in corresponding regions (Mb). Pink rectangles indicate the shared hotspot regions among the six species. Chr1-Chr7: chromosome 1 - chromosome 7. FAN: *F. x ananassa*; FII: *F. iinumae*; FNI: *F. nipponica*; FNU: *F. nubicola*; FOR: *F. orientalis*; FVE: *F. vesca*

To explore the chromosomal distribution of NBS-LRR genes, we calculated the gene numbers in each region of the chromosomes. In totally, the two largest gene numbers were found in chromosome 3 (254) and chromosome 6 (235), followed by in chromosome 5 (168) and the chromosome 7 (158), and then in chromosome 2 (79), chromosome 4 (70), and chromosome 1 (65). Although the gene number of NBS-LRRs was partly linked with chromosome lengths, the uneven distribution and the locational preference of *Fragaria* NBS-LRR genes were also found within the same chromosome or between the different chromosomes. For example, chromosome 3 has a crest region containing 19 genes, but it still has none gene in several regions; chromosome 6 displays a peak with 16 genes, while the peak of chromosome 1 is only five (Fig. 4).

Based on the locational preference of NBS-LRR genes, similar distributions were found on the same chromosomes among the different *Fragaria* species, demonstrating that the different species shared most of the highs and lows of gene numbers on each chromosome. The Duncan’s test also detected the hotspot regions shared by different species on each chromosome, which are regions with significantly higher gene numbers than other regions within the same chromosomes (*P* < 0.05). As shown in Fig. 4, except chromosome 7, the other six chromosomes have one to three shared hotspot regions of NBS-LRR genes. Interestingly, there are two, two and one shared hotspot regions of NBS-LRR genes on the ends of chromosome 3, 6 and 2, respectively, which illustrated that 39%, 26% and 30% of the NBS-LRR genes experienced gene duplication events on telomeric areas or near telomeres.

### Expression profiles of differentially expressed NBS-LRR genes after infection of powdery mildew

**Fig. 5:**
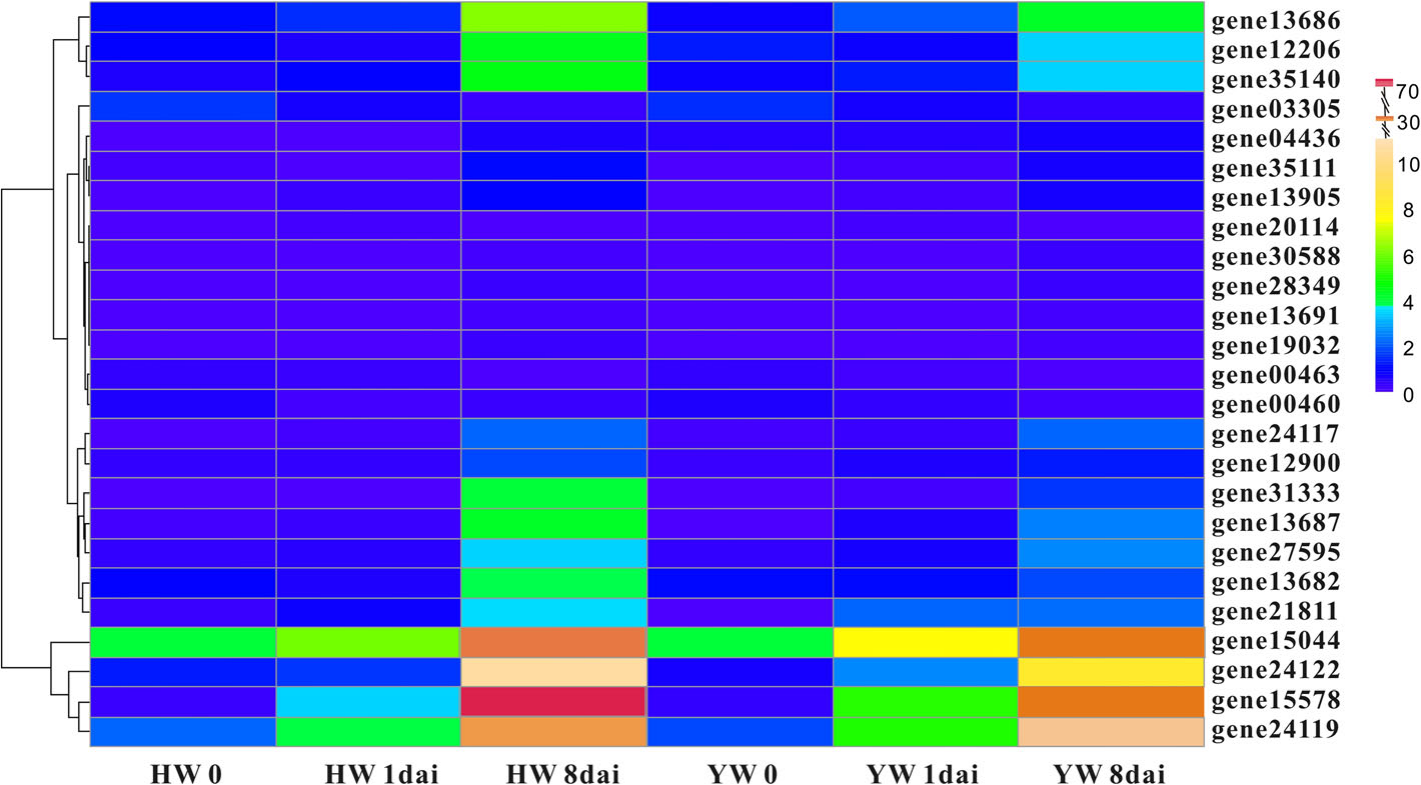
Heatmap of differentially expressed NBS-LRR genes in two *F. vesca* accessions (Hawaii 4 and Yellow Wonder 5AF7) after infection with powdery mildew. HW: Hawaii 4; YW: Yellow Wonder 5AF7; HW 0 & YW 0 mean control groups; “dai” represents day after infection. The scale bar means expression levels, represented by Fragments per Kilobase of transcript per Million mapped fragments (FPKM) value

Among all 144 NBS-LRR genes of *F. vesca*, we screened 25 NBS-LRR genes exhibiting differentially expression based on the RNA-seq data from two *F. vesca* accessions, Hawaii 4 (HW) and Yellow Wonder 5AF7 (YW), after infection with powdery mildew [30]. Although different NBS-LRR genes showed different expression levels in the two accessions, the same genes displayed similar expression pattern between the two accessions (Fig. 5). For example, gene24119 showed continuous up-regulation in both HW and YW; gene00463 manifested sustainable down-regulation; and gene12206 exhibited slight down-regulation first and then, obvious up-regulation during the infection processes in the two accessions. However, in general, the same genes had higher expression levels in HW than those in YW, such as, the expression levels of gene15578 in HW 8dai vs. YW 8dai.

Twenty-two NBS-LRR genes were up-regulated and only three of them were down-regulated genes, which indicated these NBS-LRR genes might participate in the response to infection of *P. aphanis*. Among the 22 up-regulated NBS-LRR genes, 14 of them exhibited increased expression levels both in HW and YW, and eight genes up-regulated only in HW. Interestingly, four up-regulated genes both in HW and YW includes gene15044, gene24122, gene15578, and gene24119 (Fig. 5), exhibiting prominent up-regulated expression levels compared with other genes. The three genes (gene15044, gene24122, and gene24119) with a certain amount of expression levels in controls (HW 0 and YW 0), and then showed steadily up-regulation to high expression levels. In contrast, gene15578 had very low expression in control groups, but very high expression in HW 8dai and YW 8dai, and its expression quantity in HW 8dai was the highest one among all the NBS-LRR genes from the two accessions.

### Expression profiles of differentially expressed NBS-LRR genes after infection with *P. cactorum*

**Fig. 6:**
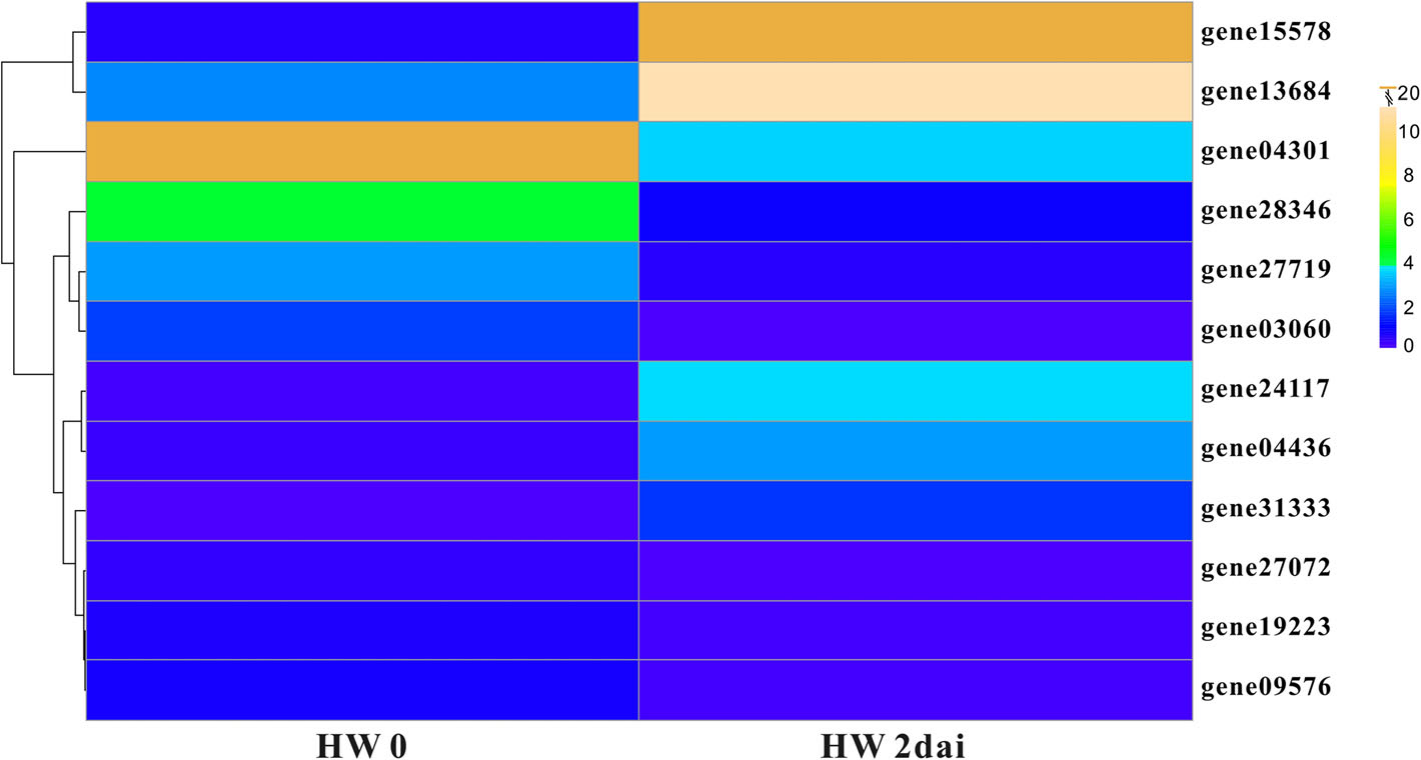
Heatmap of differentially expressed NBS-LRR genes in *F. vesca* Hawaii 4 after infection with *Phytophthora cactorum*. HW: Hawaii 4; HW 0 means control group; “dai” represents day after infection. The scale bar means expression levels, represented by Fragments per Kilobase of transcript per Million mapped fragments (FPKM) value

According to the transcriptome data of *F. vesca* Hawaii 4 infected with *P. cactorum* [31], 12 NBS-LRR genes were considered as DEGs, including five up-regulated genes and seven down-regulated genes (Fig. 6). Among the 12 genes, most of them had already exhibited different levels of expression in control group (HW 0), and then showed various expression levels in HW 2dai. For example, the up-regulated gene15578 and gene13684 had baseline expression levels in HW 0 and then showed the two highest expression levels in HW 2dai compared with other genes; and in down-regulated genes, gene04301 had the highest expression level in HW 0, then it decreased to a relative low expression level in HW 2dai. Interestingly, three of the five up-regulated genes also displayed differentially up-regulated expression after infection with powdery mildew (Figs. 5 and 6).

## Discussion

### Lineage-specific duplication driven expansion of NBS-LRRs before divergence of six *Fragaria* species

Plant NBS-LRR genes are numerous owing to a large amount of gene duplications in various genomes [15–17, 35]. Lineage-specific duplications play an important role in amplification and divergence of NBS-LRR genes in the Fabaceae, Solanaceae and Asteraceae [3, 36, 37], which were also detected in the NBS-LRR genes of the six *Fragaria* genomes, resulting in an increase of a gene family co-occurring in two or more close relatives from the common ancestor. This phenomenon was evidenced by in the phylogenetic tree that demonstrate that most of the NBS-LRR genes duplicated in the common ancestor genome of the six species, and then retained along with a small number of species-specific duplications after the speciation of the six species, because the young genus *Fragaria* originated from 1.0–4.1 MYA and the very close relationship between *Fragaria* species [38]. However, species-specific duplication principally contributed to NBS-LRR gene expansion in five Rosaceae species [14]. This difference is attributed to the relatively longer genetic distance between strawberry (*F. vesca*) and the four Rosaceae species (apple, pear, peach and mei), but the six *Fragaria* species are close relatives with very recent origin dates [38]. Therefore, the NBS-LRR genes are largely attributed to the lineage-specific duplication events in denomination of six closely related *Fragaria* species.

Moreover, most of the peak and valley values of NBS-LRR gene numbers were similar on the same chromosomes between the different species (Fig. 4), especially the shared hotspot regions of NBS-LRR genes. The results further support the fact that these genes underwent lineage-specific duplications in the common ancestor of the six *Fragaria* plants. Although introgression occurred between the *Fragaria* genomes, the high levels of conserved colinearity and macrosynteny between diploid and octoploid strawberries retained the hotspot NBS-LRR genes in the corresponding regions during the polyploidization from diploid to octoploid *Fragaria* [39].

In addition, the very high average identities were detected in TNL gene families and non-TNL gene families in all six *Fragaria* genomes, further illustrating that the close genetic relationships between the six species leading to the relative less divergence events between *Fragaria* NBS-LRR genes after gene duplications. The very significantly higher identities between orthologs than those between paralogs in both TNLs and non-TNLs (*t*-test, *P* < 0.01) suggest that more orthologs preferred to locate in NBS-LRR multi-gene families than paralogs. Furthermore, the *Ks* value between paralogs or orthologs, which is the molecular clock of duplication time of genes in one species, or the divergence time of different species, respectively [40], revealed that the paralogous genes had highly significantly greater *Ks* and *Ka*/*Ks* values than those of orthologous genes in NBS-LRRs (*P* < 0.01), manifesting that paralogs evolved faster than orthologs, and the NBS-LRR genes are under faster evolutionary processes intra-species instead of inter-species among the six *Fragaria* genomes. Thus, most of the duplication events of *Fragaria* NBS-LRRs were lineage-specific duplications which occurred before the divergence of the six *Fragaria* species.

### Distinct evolutionary histories between TNL and non-TNL gene families

Plant NBS-LRR genes are believed to share a common ancestor with ancient origination, which could be classified into two major types, TNLs and non-TNLs, according to the presence of the TIR, CC or X in the N-terminal domains [41].

The TNL and non-TNL genes located separately in phylogenetic tree constructed by the NBS domains in the *Fragaria* genomes (Additional file 3: Figure S1), legume family and other plants [3, 14, 32, 42]. In addition, TNL and non-TNL genes differ in terms of the topologies of phylogenetic analysis, especially the distinct branch lengths between the two type genes. Previous studies have revealed that the branch lengths between TNL genes were significantly longer than those between non-TNLs in *A. thaliana* and *A. lyrata* [19]. Here, branches were slightly longer in TNLs than those in non-TNLs, indicating that TNL genes might evolve faster than non-TNLs on the whole. More complex phenomena were uncovered that the phylogenetic tree of TNL genes had two distinct groups (group I and II). The branch lengths of non-TNLs were significantly longer than those in TNL group I and significantly shorter than TNL group II (*P* < 0.01), which manifested the different evolutionary patterns between the TNL and non-TNL genes.

The duplication of NBS-LRR genes in the six *Fragaria* genomes also provided opportunities to detect the evolutionary rates between TNL and non-TNL genes. The diversity and *Ks* values of TNL duplicates were significantly higher than those of non-TNLs (*P* < 0.01), consistent with the previous studies in *Arabidopsis*, soybean, and apple genomes [14, 19, 43], which might suggest that there are different evolutionary pattern between the TNLs and non-TNLs.

Our results demonstrated that TNL genes were under stronger selective pressures compared with non-TNLs, which were also reported in *Arabidopsis* relatives, five Rosaceae plants and soybean genomes [14, 19, 43]. However, the opposite results were found that *Ka*/*Ks* ratios were lower in TNLs than in non-TNL genes from poplar, etc. [16, 37]. The contrary phenomena on evolution of TNLs and non-TNLs might be due to different plants growing in diverse environments along with different life cycles and developmental conditions [14]. Therefore, TNLs and non-TNLs may have diverse evolutionary patterns to adapt to their corresponding pathogens in specific environments.

### *R* genes differentially respond to pathogens with different genetic backgrounds

Presentations of different responses to the same pathogens were commonly found in genetically heterogeneous accessions or varieties in strawberries [44–46]. For *P*. *aphanis* fungus infecting two *F. vesca* accessions, the white mycelia appear earlier and faster on the leaf of susceptible accession YW compared with the less-susceptible HW, and more up-regulated and down-regulated genes in HW than YW [30]. Therefore, although similar expression profiles of the NBS-LRR genes between the two *F. vesca* ecotypes (HW and YW), the less-susceptible HW exhibited higher expression levels and more pathogen-involved NBS-LRR genes than the susceptible YW (Fig. 5). The results indicated that the responses of *R* genes were stronger in less-susceptible accession than the susceptible one, which were consistent with previous studies on expression profiles of NBS-LRRs after infection with strawberry pathogens. Responses to *Colletotrichum* infection, for example, the FvNBSs manifested ecotype-specific responses between the moderately resistant ectype YW and the susceptible ecotype HLJ [44]; the response of genes was quicker and/or stronger in the moderate resistance cultivar ‘Andana’ than in the susceptible cultivar Camarosa [45]. The transcriptional responses of NBS-LRR genes showed more sensitive and fast-growing expression levels in less-susceptible cultivar ‘Sweet Charlie’ compared with those in susceptible ‘Jiuxiang’ [47]. For strawberry infection with *P. cactorum*, the NBS genes responded more quickly and strongly in the resistance genotype ‘Bukammen’ than in the susceptible FDP821 [46].

More interestingly, although *R* genes manifested different response to the same pathogen with different genetic backgrounds, the *R* gene might display similar expression pattern after different pathogen infections. Three NBS-LRR genes (gene15578, gene24117 and gene31333) always had up-regulated expression in *F. vesca* after infection both by powdery mildew and *P. cactorum*. Especially, the gene15587 possessed the highest expression levels with powdery mildew infection and *P. cactorum* infection, suggesting that the same *R* gene might participate in response to different pathogens, as reported in other plants [48]. For example, *Arabidopsis* activated disease resistance gene 1 (*ADR1*) encoding special CC-NBS-LRR proteins (CCR-NBS-LRR), participates in host-cell defense responses to *Peronospora parasitica* and *Erysiphe cichoracearum* [48].

Furthermore, most of the DEGs were located in the *Fragaria* lineage-specific duplicated clades in the phylogenetic tree (Additional file 3: Figure S1). Among these DEGs, gene31333, gene04436 and gene00460 had *Ka*/*Ks* ratios larger than 1, indicating positive selection acting on these *R* genes. All of these might provide chances for screening of functional genes or molecular markers related to disease-resistance in strawberry genomes.

## Conclusions

A total of 1134 NBS-LRRs were identified in the six *Fragaria* species, including 325, 155, 190, 187, 133 and 144 NBS-LRRs in *F. x ananassa*, *F. iinumae*, *F. nipponica*, *F. nubicola*, *F. orientalis*, and *F. vesca*, respectively. Among the NBS-LRR genes, 866 of them could be classified into 184 multi-gene families across the six *Fragaria* genomes, with highly significantly greater identities in orthologs than those in paralogs. In contrast, the *Ks* ratios of orthologs were extremely significantly lower than those of paralogs in all NBS-LRR multi-gene families. There were more *Fragaria* lineage-specific duplicated clades with short branch lengths and shallow nodes than species-specific duplicated clades in the phylogenetic tree. The shared hotspot regions of duplicated NBS-LRRs were detected on the same chromosomes across the six *Fragaria* species. All of these results suggest lineage-specific duplications of NBS-LRR genes occurred before the divergence of the six *Fragaria* species. In addition, the TNLs had significantly greater *Ks* and *Ka*/*Ks* ratios than non-TNLs, demonstrating that the TNLs duplicated earlier with more rapid evolutionary rate and under stronger selective pressures than non-TNLs. Furthermore, the expression patterns of NBS-LRR genes indicated that the same *R-*gene showed different expression profiles under different genetic backgrounds in response to pathogens.

## Additional files

Additional file 1: Table S1. Number of NBS-LRR genes in each family among six *Fragaria* species. (XLSX 16 kb)

Additional file 2: Table S2. Identities of NBS-LRR genes in TNL and non-TNL gene families. (XLSX 66 kb)

Additional file 3: Figure S1. Phylogenetic tree of all NBS-LRR genes among six *Fragaria* species. The red, yellow, purple, light blue, green and blue circles represent genes from *F. x ananassa*, *F .iinumae*, *F. nipponica*, *F. nubicola*, *F. orientalis* and *F. vesca*, respectively. The red circle means lineage-specific duplicated clades and the red rectangle means species-specific duplicated clades. DEG in HW and YW after infection with powdery mildew is marked by yellow highlight; DEG in HW during infection with *P. cactorum* is marked by blue wave line. (PDF 281 kb)

Additional file 4: Figure S2. Species tree of the six *Fragaria* species. (JPEG 23 kb)

Additional file 5: Figure S3. The *Ks* ranges of NBS-LRR genes in six *Fragaria* species. The *Ks* ranges between paralogs (A), orthologs (B) and all genes (C) in TNLs and the *Ks* ranges between paralogs (D), orthologs (E) and all genes (F) in non-TNLs among the six species. (JPEG 1759 kb)

## Abbreviations

CDS: Nucleotide coding sequences
NBS-LRR: Nucleotide binding sites-leucine-rich repeats
R gene: Resistance gene
