# Supplementary figures and images for: Lineage-specific duplications of NBS-LRR genes occurring before the divergence of six Fragaria species
Source: BMC Genomics. 2018 Feb 8;19:128. doi: 10.1186/s12864-018-4521-4 (PMC5806312; doi:10.1186/s12864-018-4521-4)

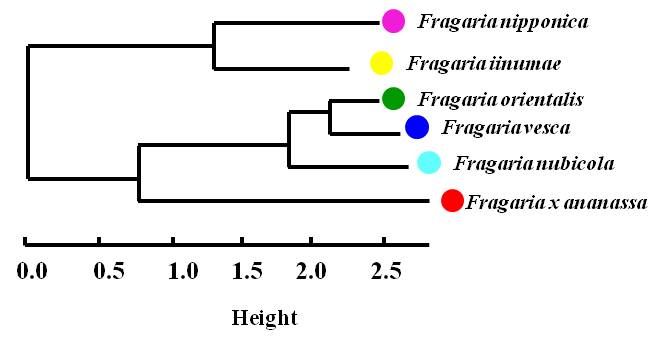

Supplement: Supplementary file 4 — Species tree of the six Fragaria species. (JPEG 23 kb) [file 12864_2018_4521_MOESM4_ESM.jpg]

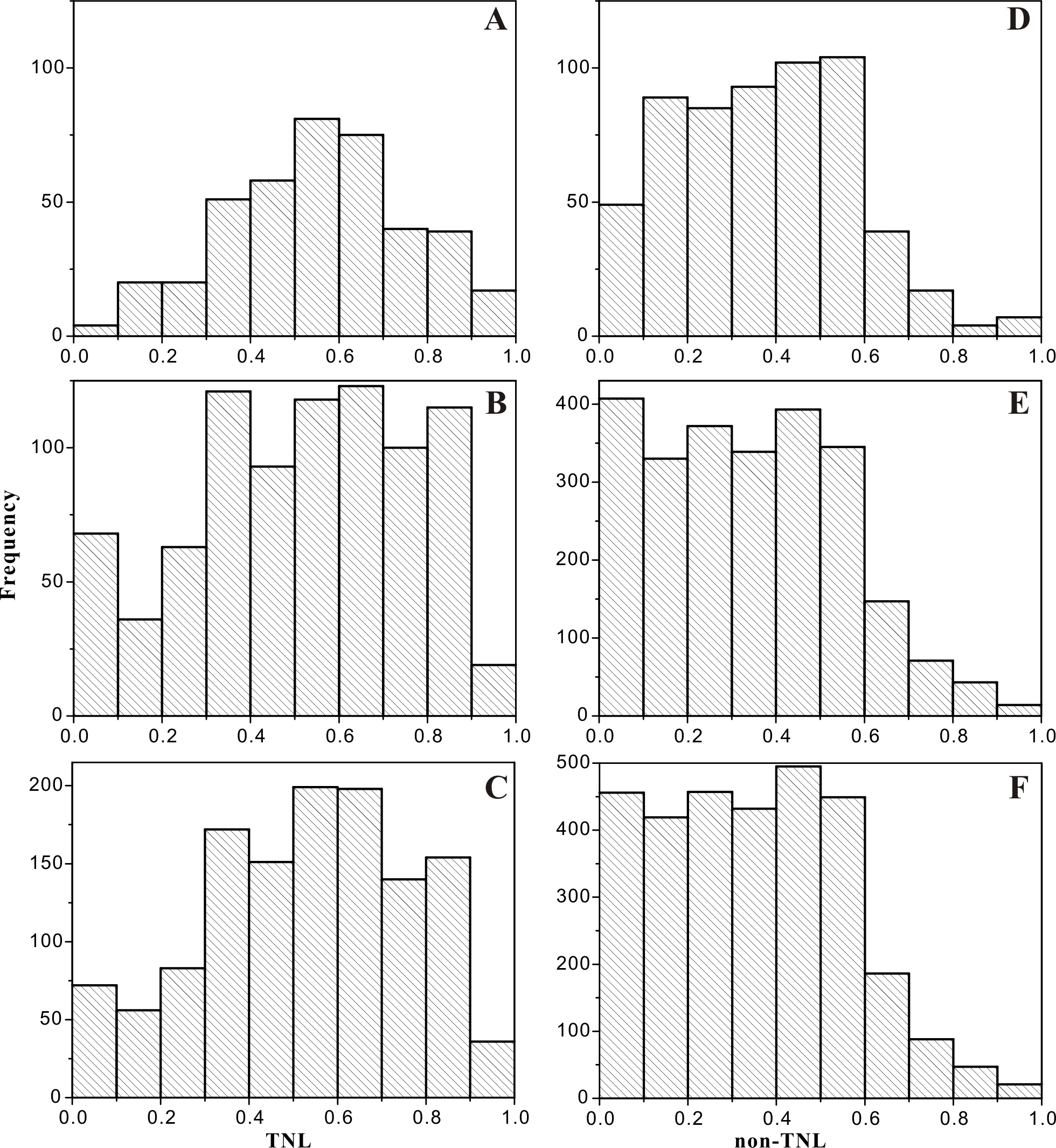

Supplement: Supplementary file 5 — The Ks ranges of NBS-LRR genes in six Fragaria species. The Ks ranges between paralogs (A), orthologs (B) and all genes (C) in TNLs and the Ks ranges between paralogs (D), orthologs (E) and all genes (F) in non-TNLs among the six species. (JPEG 1759 kb) [file 12864_2018_4521_MOESM5_ESM.jpg]
