# Supplementary material for: Lineage-specific duplications of NBS-LRR genes occurring before the divergence of six Fragaria species
Source: BMC Genomics. 2018 Feb 8;19:128. doi: 10.1186/s12864-018-4521-4 (PMC5806312; doi:10.1186/s12864-018-4521-4)

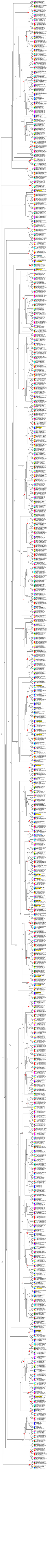

Supplement: Supplementary file 3 — Phylogenetic tree of all NBS-LRR genes among six Fragaria species. The red, yellow, purple, light blue, green and blue circles represent genes from F. x ananassa, F .iinumae, F. nipponica, F. nubicola, F. orientalis and F. vesca, respectively. The red circle means lineage-specific duplicated clades and the red rectangle means species-specific duplicated clades. DEG in HW and YW after infection with powdery mildew is marked by yellow highlight; DEG in HW during infection with P. cactorum is marked by blue wave line. (PDF 281 kb) [file 12864_2018_4521_MOESM3_ESM.pdf]
